# Brain tumor segmentation using multi-scale attention U-Net with EfficientNetB4 encoder for enhanced MRI analysis

**DOI:** 10.1038/s41598-025-94267-9

**Published:** 2025-03-22

**Authors:** Preetha R, Jasmine Pemeena Priyadarsini M, Nisha J S

**Affiliations:** School of Electronics Engineering, Vellore Institute of Technology, Vellore, 632014 Tamilnadu India

**Keywords:** Brain tumor, Deep learning (DL), EfficientNet, Magnetic resonance imaging (MRI), U-Net, Segmentation, Health care, Medical research, Oncology, Engineering

## Abstract

Accurate brain tumor segmentation is critical for clinical diagnosis and treatment planning. This study proposes an advanced segmentation framework that combines Multiscale Attention U-Net with the EfficientNetB4 encoder to enhance segmentation performance. Unlike conventional U-Net-based architectures, the proposed model leverages EfficientNetB4’s compound scaling to optimize feature extraction at multiple resolutions while maintaining low computational overhead. Additionally, the Multi-Scale Attention Mechanism (utilizing $1\times 1, 3\times 3$, and $5\times 5$ kernels) enhances feature representation by capturing tumor boundaries across different scales, addressing limitations of existing CNN-based segmentation methods. Our approach effectively suppresses irrelevant regions and enhances tumor localization through attention-enhanced skip connections and residual attention blocks. Extensive experiments were conducted on the publicly available Figshare brain tumor dataset, comparing different EfficientNet variants to determine the optimal architecture. EfficientNetB4 demonstrated superior performance, achieving an Accuracy of 99.79%, MCR of 0.21%, Dice Coefficient of 0.9339, and an Intersection over Union (IoU) of 0.8795, outperforming other variants in accuracy and computational efficiency. The training process was analyzed using key metrics, including Dice Coefficient, dice loss, precision, recall, specificity, and IoU, showing stable convergence and generalization. Additionally, the proposed method was evaluated against state-of-the-art approaches, surpassing them in all critical metrics, including accuracy, IoU, Dice Coefficient, precision, recall, specificity, and mean IoU. This study demonstrates the effectiveness of the proposed method for robust and efficient segmentation of brain tumors, positioning it as a valuable tool for clinical and research applications.

## Introduction

The brain, a critical central nervous system component, governs various bodily functions through a complex network of neurons. Any irregular growth or disruption within brain cells can lead to significant functional impairments in associated organs^1^. Brain tumors, which result from the uncontrolled growth of brain cells, represent a major public health challenge. According to the American Cancer Society, approximately 25,400 new cases of malignant brain and spinal cord tumors are expected to be diagnosed in the United States in 2023, comprising 14,420 cases in men and 10,980 cases in women^2^. If benign tumors were also included, the number would rise considerably. Furthermore, it is estimated that around 18,760 individuals will lose their lives to these malignancies, including 10,690 males and 8,070 females. Brain tumors are ranked as the tenth leading cause of cancer-related deaths in the United States^3^.

Brain tumors are life-threatening abnormalities characterized by the rapid growth of abnormal tissue within or around the brain, posing significant challenges to human health due to their complex nature and potential for severe neurological damage^4^. These tumors, classified as benign or malignant, often require early and accurate detection to optimize treatment outcomes and enhance survival rates^5^. Gliomas contribute to most malignant brain tumors, and glioblastoma multiforme (GBM) is the most aggressive and has a terrible prognosis^6^. Effective treatment planning demands a precise diagnosis and exact segmentation of brain tumors from imaging data^7^.

The diagnosis of brain tumors is mainly based on medical imaging. Computed tomography (CT) and magnetic resonance imaging (MRI) scans are crucial for diagnosing and categorizing brain cancers. Accurate detection and subsequent medical planning are made possible by these imaging approaches^8^. Since MRI generates images using radio waves and a powerful magnetic field without exposing patients to ionizing radiation like X-rays, it is recommended over CT scans. MRI offers highly detailed visualizations of the body’s internal structures, particularly soft tissues. It generates a range of high-contrast grayscale images, such as T1 contrast-enhanced, FLAIR, T2, T1-weighted, and Proton Density (PD) images, each of which provides a distinct viewpoint on the properties and anomalies of the tissue. However, the gold standard for brain imaging, manual segmentation of magnetic resonance imaging (MRI) data, is a labor-intensive procedure that is prone to inter-observer variability, rendering it unreliable and inefficient for routine clinical use^9^.

To address these challenges, automated segmentation techniques based on artificial intelligence (AI) have gained significant attention in medical image analysis^10^. Among these, convolutional neural networks (CNNs)^11^ have demonstrated remarkable potential in medical imaging tasks by learning hierarchical features directly from data^12^. The U-Net architecture, widely adopted for biomedical image segmentation, has proven effective due to its symmetric encoder-decoder structure and skip connections, which facilitate the integration of high-level semantic information and low-level spatial details^13^. Despite its widespread use, standard U-Net struggles to handle datasets with high variability in tumor size, shape, intensity, and imbalanced class distributions, limiting its effectiveness in challenging cases.

The U-Net architecture’s ability to perform difficult segmentation tasks could be significantly enhanced by combining advanced methods of feature extraction and attention mechanisms^14^. Attention mechanisms help the model in precisely identifying complex tumor borders by reducing irrelevant information and concentrating on key areas of interest^15^. EfficientNet provides strong feature extraction through compound scaling, which maintains the ideal balance between network resolution, depth, and width^16^. The most sophisticated version, EfficientNetB4, is ideal for medical image segmentation because it offers excellent performance while preserving computational efficiency^17^.

To overcome the challenges that exist with traditional U-Net models in brain tumor segmentation, this work proposes an enhanced U-Net architecture with a multi-scale attention mechanism using EfficientNetB4 as the encoder. By acquiring information across multiple spatial scales, the multi-scale attention mechanism enables the model to accurately capture features from tumors of different sizes and complexity^18^. Also, the EfficientNetB4 backbone increases the model’s feature extraction capabilities, promising strong segmentation results. When tested on publicly available Figshare MRI brain tumor datasets, the proposed method surpasses existing approaches in key metrics such as recall, precision, Dice Coefficient, and IoU.The proposed method strengthens the precision, effectiveness, and reliability of automated medical image analysis by addressing the difficulties in brain tumor segmentation, thereby contributing to better clinical decision-making and treatment planning.

Brain tumor segmentation plays a critical role in clinical decision-making, including surgical planning, radiotherapy targeting, and monitoring tumor progression. Accurate and reliable segmentation is essential for improving patient outcomes. Despite significant progress in deep learning approaches for brain tumor segmentation, several challenges remain that hinder clinical deployment, such as variability in MRI acquisition protocols, generalizability across patient populations, and integration into clinical workflows. Recent studies have achieved remarkable segmentation performance using advanced neural network architectures. However, many of these approaches face limitations in clinical application readiness due to the lack of external validation, explainability, and adaptability to diverse clinical settings. Moreover, the complexity and computational demands of state-of-the-art models pose challenges for real-time clinical use. This study addresses these limitations by proposing an enhanced U-Net architecture with a multi-scale attention mechanism using EfficientNetB4 as the encoder.

Our key contributions include:

- This study introduces a novel multi-scale attention-based U-Net architecture that integrates EfficientNetB4 as the encoder backbone. The innovative use of multi-scale convolutions ($1\times 1, 3\times 3, 5\times 5$) enhances feature extraction by capturing fine-grained details and global contextual information, significantly improving tumor boundary delineation in MRI segmentation.
- EfficientNetB4’s compound scaling strategy optimally balances model depth, width, and resolution, maintaining high-resolution feature maps while minimizing computational cost. This enables better hierarchical feature extraction, crucial for segmenting complex tumor regions.
- An extensive comparison is conducted on the performance of EfficientNet variants (B0 to B7) for brain tumor segmentation. EfficientNetB4 is identified as the optimal balance between accuracy and computational efficiency.
- Unlike existing methods that heavily depend on data augmentation, this approach achieves high segmentation accuracy using only standard preprocessing techniques (CLAHE, Gaussian blur, and intensity normalization). This demonstrates the model’s inherent generalization ability across different tumor structures and MRI modalities.
- A detailed visual comparison of original images, ground truth masks, and predicted segmentation masks is provided, demonstrating the model’s capability to accurately delineate tumor boundaries.
- Our proposed model’s performance is evaluated using comprehensive metrics including Dice Similarity Coefficient (DSC), Intersection over Union (IoU), Mean IoU, Accuracy, Precision, Recall, Specificity, and Misclassification Rate (MCR). These metrics provide a thorough assessment of the segmentation quality and model performance
- Beyond academic evaluation, the study explores the clinical deployment potential of the proposed model, including regulatory challenges and integration into real-world radiology settings. EfficientNet-B4’s computational efficiency makes it suitable for deployment on resource-constrained medical devices.

These contributions advance the field of brain tumor segmentation by providing a robust, efficient, and clinically relevant solution for MRI image analysis.

The remainder of this paper is structured as follows: “Related work” reviews related work in the field of brain tumor segmentation using DL models. “Proposed workflow” details the proposed workflow, including preprocessing techniques, U-Net architecture, EfficientNetB4 architecture, design of the multi-scale attention U-Net with EfficientNetB4 encoder and mathematical formulation details. In “Experimental setup”, we describe the experimental setup, covering dataset, implementation details, hyper parameter values and performance evaluation metrics. “Results and discussions” presents the results obtained from the experiments, followed by a comprehensive discussion of the findings, their implications, and a comparison with state-of-the-art methods. “Conclusion” concludes the paper, “Limitations” presents the limitations, and finally “Future work” describes potential areas for future research.

## Related work

Brain tumor segmentation has gained significant attention due to its critical role in medical imaging and its potential to improve clinical decision-making. Early developments in this field were led by Shelhamer et al.^19^, who introduced Fully Convolutional Networks (FCNs), laying the groundwork for image segmentation tasks, including brain tumor segmentation. These innovations provided the initial framework for applying deep learning techniques in medical imaging, which later evolved into more specialized and advanced architectures. The introduction of U-Net, a model created especially for biomedical image segmentation, by Ronneberger et al.^20^ was a breakthrough contribution. U-Net became a standard for medical imaging segmentation tasks because of its unique encoder-decoder architecture with skip connections, allowing for effective feature extraction while maintaining spatial details. Iqbal et al.^21^ extended deep convolutional neural network (DCNN) architecture for accurate brain tumor segmentation in MRI images, leveraging the BRATS 2015 dataset’s multimodal images to demonstrate superior performance.

Researchers have investigated several architectural innovations over time to resolve the challenges associated with brain tumor segmentation, such as overlapping intensities and unclear boundaries. Hussain et al.^22^ developed a new ILinear nexus DCNN architecture that uses parallel and linear layer arrangements, two-phase weighted training, and advanced preprocessing and postprocessing techniques to achieve high performance. Cui et al.^23^ developed a cascaded DL neural network that combines a tumor localization network (TLN) and an intratumor classification network (ITCN) for precise and efficient brain tumor segmentation and subregion classification. To enhance segmentation accuracy, attention mechanisms and residual learning were also employed. Verma et al.^24^ proposed RR-U-Net,a U-Net-based architecture enhanced with residual modules achieving high DSC scores on widely used datasets. This approach demonstrated the effectiveness of residual blocks for capturing intricate tumor patterns. Similarly, Kumar et al.^25^ incorporated residual connections in U-Net for brain tissue segmentation using FLAIR MRI images, achieving better segmentation accuracy but adding computational complexity.

The integration of DenseNet121 with U-Net architectures further improved feature reuse and segmentation outcomes, as demonstrated by Cinar et al.^26^, who applied this hybrid model to the BraTS dataset. However, the reduced image resolution used in their experiments limited the model’s ability to capture fine-grained tumor details, a critical factor in clinical applications. Similarly, Yadav et al.^27^ introduced EffUNet++, integrating EfficientNet-B7 as an encoder within the UNet++ framework, which demonstrated robust segmentation performance but faced challenges related to dataset dependency and pre-trained weight reliance. Researchers have also explored models combining segmentation and classification tasks. Zafar et al.^28^ proposed a hybrid approach integrating U-Net for segmentation with YOLO-based methods for tumor detection and classification, achieving high accuracy and efficiency. However, the computational requirements of this approach highlight the need for lightweight architectures capable of balancing performance with resource efficiency.

Emerging approaches also focus on leveraging multimodal imaging for enhanced segmentation. Guo et al.^29^ emphasized the potential of combining data from different imaging modalities to improve tumor delineation. However, challenges such as misaligned data and the need for robust frameworks to process diverse datasets remain unresolved. Aboussaleh et al.^30^ addressed these issues by proposing a hybrid 3DUV-NetR+ model combining U-Net, V-Net, and Transformer architectures for 3D MRI data, demonstrating improved spatial feature extraction.

Recent advancements in brain tumor segmentation have significantly improved the accuracy and efficiency of automated diagnostic tools. A comprehensive review of advanced techniques highlights the effectiveness of cascaded networks, ensembling methods, and federated learning approaches for enhanced segmentation accuracy and privacy preservation^31^. One study introduced a customized 3D U-Net model for multiclass brain tumor segmentation using volumetric images from the BraTS 2020 dataset, achieving high Dice scores for enhancing tumor, tumor core, and whole tumor regions^32^. Another research proposed a deep learning ensemble integrating UNet3D, V-Net, and MSA-VNet models, which outperformed individual models in segmenting gliomas^33^. The use of mutual enhancing networks (MENs) for fully automatic segmentation of brain tumor subregions has also shown promise, with a unique network architecture incorporating retina U-Net and a classification-localization module^34^. The studies^35–40^ collectively demonstrate the potential of advanced deep learning architectures, transfer learning, and explainable AI in improving brain tumor segmentation accuracy and generalizability across different datasets.

Several studies have demonstrated the effectiveness of transfer learning and customized architectures in achieving high accuracy. A study employing five pre-trained EfficientNet variants (B0-B4) for multi-class brain tumor classification achieved high accuracy, precision, recall, and F1-score using EfficientNetB2 on the CE-MRI Figshare dataset^41^. Another research proposed a customized pre-trained EfficientNetB7 model, achieving high accuracy and MIOU on a public dataset^42^. A pre-trained EfficientNetB4 model with adjustable learning rate and custom callbacks achieved 99.67% accuracy on the Br35h dataset and 99.87% accuracy on an augmented version^43^. In the realm of segmentation, a spatial-attention-enhanced U-Net model achieved a Dice similarity coefficient of 0.93 on the Figshare dataset, outperforming methods like V-Net and DeepLab V3+^44^. These studies collectively demonstrate the potential of transfer learning, attention mechanisms, and residual learning in improving brain tumor detection and segmentation accuracy.

Despite these advancements, limitations such as insufficient annotated datasets, high computational demands, and challenges in fine-grained segmentation persist. Lightweight architectures like those proposed by Walsh et al.^45^ offer promising solutions by reducing training data requirements, though further research is needed to address tumor core and edema segmentation challenges effectively. Overall, the evolution of DL models for brain tumor segmentation has showcased significant progress, transitioning from traditional CNNs to sophisticated architectures like U-Net derivatives, DenseNet hybrids, and Transformer-based models. However, future research must address challenges related to generalization across diverse datasets, efficient processing of multimodal imaging, and computational scalability to fully harness the potential of these models in clinical settings. Table 1 shows comparison of brain tumor segmentation models on Figshare dataset.

**Table 1:** Comparison of brain tumor segmentation models on Figshare dataset.

Reference	Model/approach	Dataset	Key features and limitations
Sahoo et al. (2023)	Residual U-Net based transfer learning model	Figshare	$\bullet$ No pre-processing used $\bullet$ Reduced feature extraction for large tumors
Mayala et al. (2022)	MST-based method	Figshare	$\bullet$ Interactive segmentation $\bullet$ Graph-based approach $\bullet$ Limited to Gaussian filter
El-Shafai et al. (2022)	Hybrid segmentation approach	Figshare	$\bullet$ Combines traditional and deep learning methods $\bullet$ Noise sensitivity and difficulty with flat images
Kasar et al. (2021)	DNNs - U-Net, SEGNET	Figshare	$\bullet$ Semantic segmentation approach $\bullet$ No separate DSC for tumor and background
Sobhaninia et al. (2020)	Cascaded Deep Neural Networks	Figshare	$\bullet$ Uses multiple image scales $\bullet$ Needs improved Dice value
Díaz-Pernas et al. (2021)	Multiscale CNN	Figshare	$\bullet$ Combines multiscale features $\bullet$ False positives from skull and vertebral parts
Razzaghi et al. (2022)	Multimodal Deep Transfer Learning	Figshare	$\bullet$ Combines multimodal data $\bullet$ Scalability issues with more modalities
Verma et al. (2024)	RR-U-Net	Figshare	$\bullet$ U-Net with residual modules $\bullet$ Lacks advanced architectures like DenseNets

In recent years, the integration of EfficientNet architectures with attention mechanisms has garnered significant interest in medical image analysis, particularly in segmentation tasks. EfficientNet, known for its compound scaling and efficient feature extraction, has been enhanced with various attention modules to improve performance. For instance, Canayaz proposed an EfficientNet variant incorporating attention blocks, demonstrating that the attention mechanism plays a critical role in extracting deep-level features^46^. Similarly, a study introduced a novel adaptation of the EfficientNetV2 architecture, enhanced with Global Attention Mechanism (GAM) and Efficient Channel Attention (ECA), aiming to improve segmentation accuracy^47^.

In the context of brain tumor segmentation, our work distinguishes itself by proposing an enhanced U-Net architecture that integrates a multi-scale attention mechanism with EfficientNetB4 as the encoder. While previous studies have explored the combination of EfficientNet with attention modules, our approach specifically addresses the challenges associated with high variability in tumor size, shape, and intensity. The multi-scale attention mechanism enables the model to capture features across different spatial scales, enhancing its ability to delineate complex tumor boundaries. Furthermore, by leveraging EfficientNetB4’s efficient feature extraction capabilities, our model achieves superior performance in segmenting brain tumors from MRI images. This targeted application and architectural innovation position our work as a significant advancement in the field, offering a robust and efficient solution for brain tumor segmentation that builds upon and extends the existing methodologies combining EfficientNet and attention mechanisms.

## Proposed workflow

**Fig. 1:**
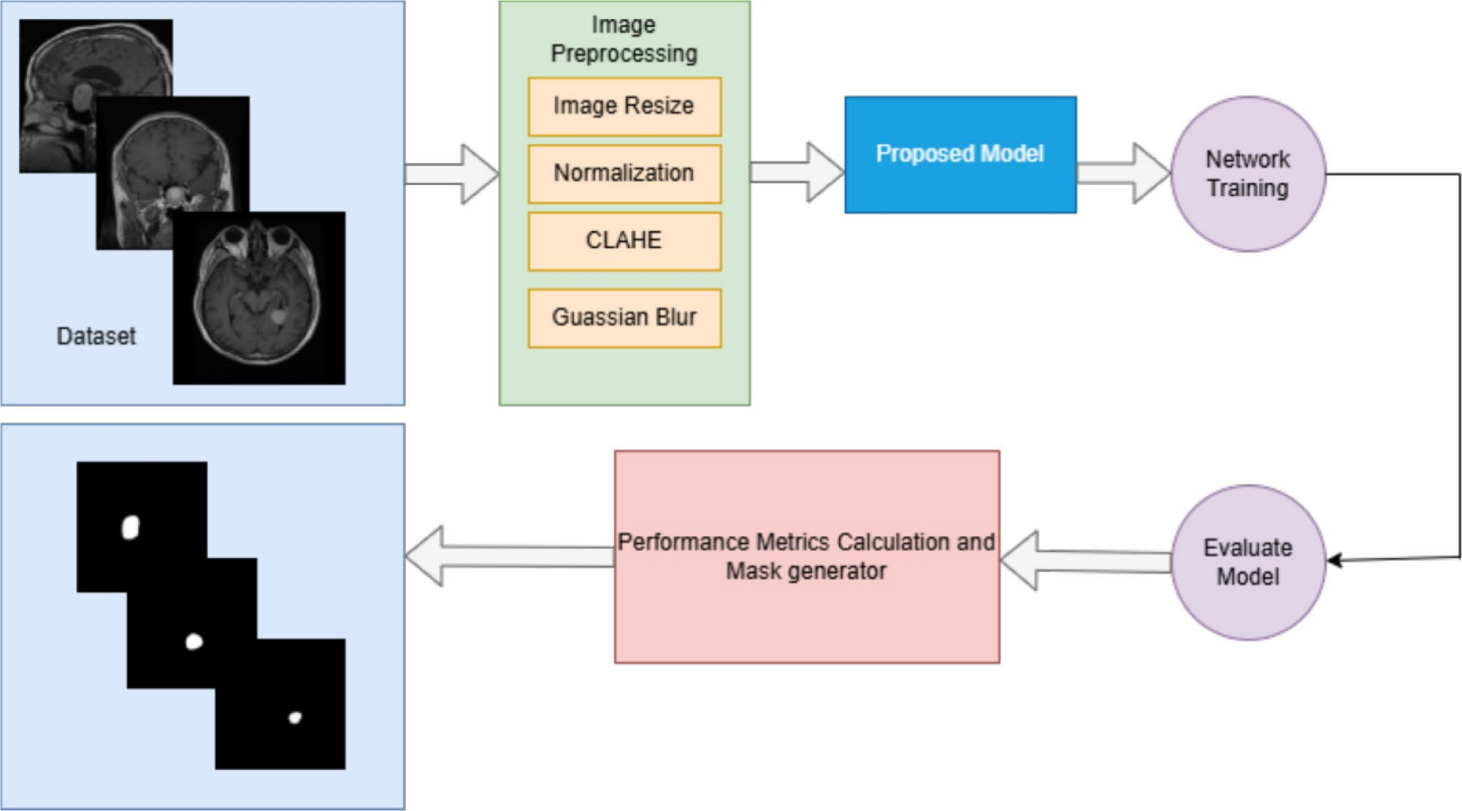
Workflow of the proposed method.

In this study, we propose a novel DL based model for brain tumor segmentation that integrates the power of an EfficientNetB4 encoder with a Multi-Scale Attention U-Net decoder. The model’s primary objective is to achieve accurate and robust segmentation of brain tumors from MRI images by combining efficient feature extraction with advanced attention mechanisms that improve the segmentation accuracy. This approach utilizes the strengths of EfficientNetB4 as a pre-trained feature extractor, followed by a decoder enhanced with multi-scale attention and residual blocks to capture better spatial hierarchies and fine-grained details relevant to tumor segmentation. The proposed model begins by passing the input MRI image through the EfficientNetB4 encoder, pre-trained on the ImageNet dataset. This encoder captures hierarchical features across multiple scales, allowing the model to learn low-level and high-level image representations. These features are then passed through a U-Net-based decoder, where skip connections from the encoder are merged with upsampled features at each decoding step. To further enhance the segmentation process, multi-scale attention blocks are employed, allowing the model to concentrate on significant areas of the image by emphasizing relevant features and suppressing irrelevant ones. Residual connections in the decoder ensure the preservation of important spatial information throughout the upsampling process. The output of the network is a binary segmentation mask, which is generated by a final 1x1 convolution layer, followed by a sigmoid activation function. This output represents the regions of the image corresponding to the brain tumor. The proposed workflow in the form of a block diagram is shown in Fig. 1. The methodology outlined below describes in detail the structure and functioning of both the EfficientNetB4 encoder and the Multi-Scale Attention U-Net decoder, highlighting the innovations introduced to optimize the segmentation performance for brain tumor detection in MRI scans.

This study primarily focuses on brain tumor segmentation rather than classification. We prioritize the accurate delineation of tumor regions in MRI scans, which is a crucial prerequisite for subsequent analysis and treatment planning. Our approach allows for potential future work in tumor classification or grading based on the segmented regions.

### Preprocessing

**Fig. 2:**
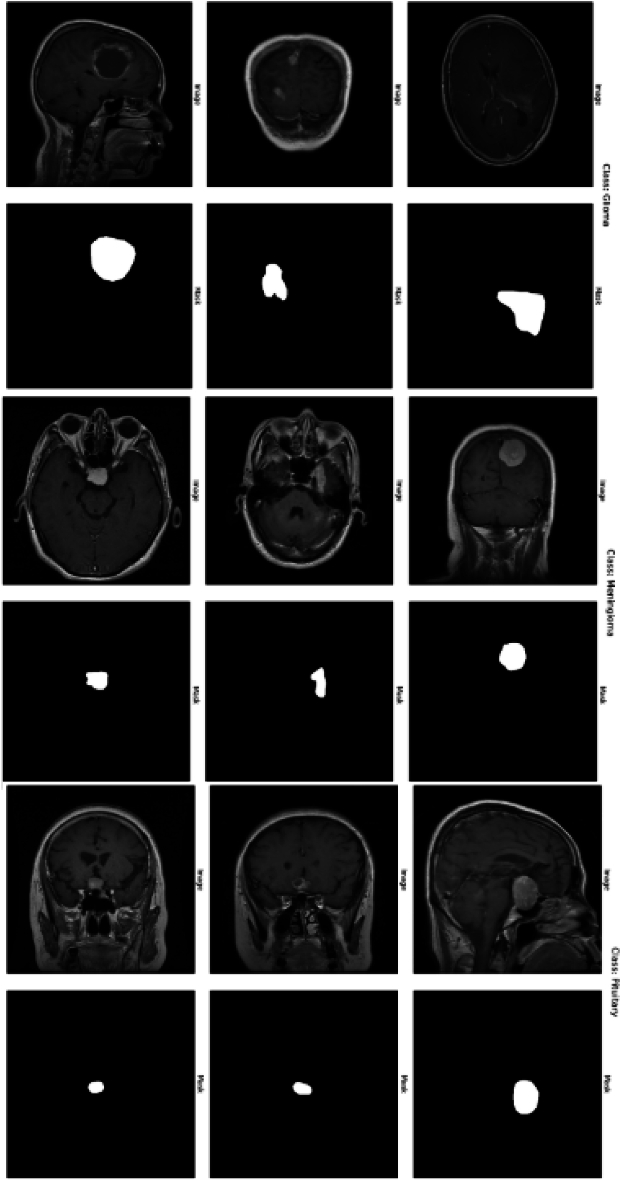
Sample MRI images and their corresponding masks in the dataset.

Before training the model, several preprocessing steps are applied to the dataset. The raw images, initially in RGB color space, are first converted to the LAB color space to apply CLAHE (Contrast Limited Adaptive Histogram Equalization) to the L-channel. This step enhances the contrast of the images, especially in areas with low intensity. After CLAHE is applied, the image is converted back to BGR color space. Gaussian blur is then applied to the images to reduce noise and smooth out the image, improving the quality of the input data. The images are then resized to a consistent dimension of 256x256 pixels and normalized by dividing by 255, transforming the pixel values to the range [0, 1]. The grayscale images are resized to 256x256 pixels for the masks, normalized, and reshaped to include a single channel. The dataset is divided into training and testing sets with 90:10 split. Specifically, 90% of the samples are allocated for training the model, while the remaining 10% are set aside for testing and evaluating the model on unseen data. Figure 2 presents sample MRI images and their corresponding masks. Class-wise distribution of the dataset and data splitting ratio is provided in Table 2.

**Table 2:** Class-wise distribution of the dataset and data splitting ratio.

Tumor type	Tumor slices	Training images	Testing images
Glioma	1426	1283	143
Meningioma	708	637	71
Pituitary tumor	930	838	92
Total	3064	2758	306

### U-Net architecture

**Fig. 3:**
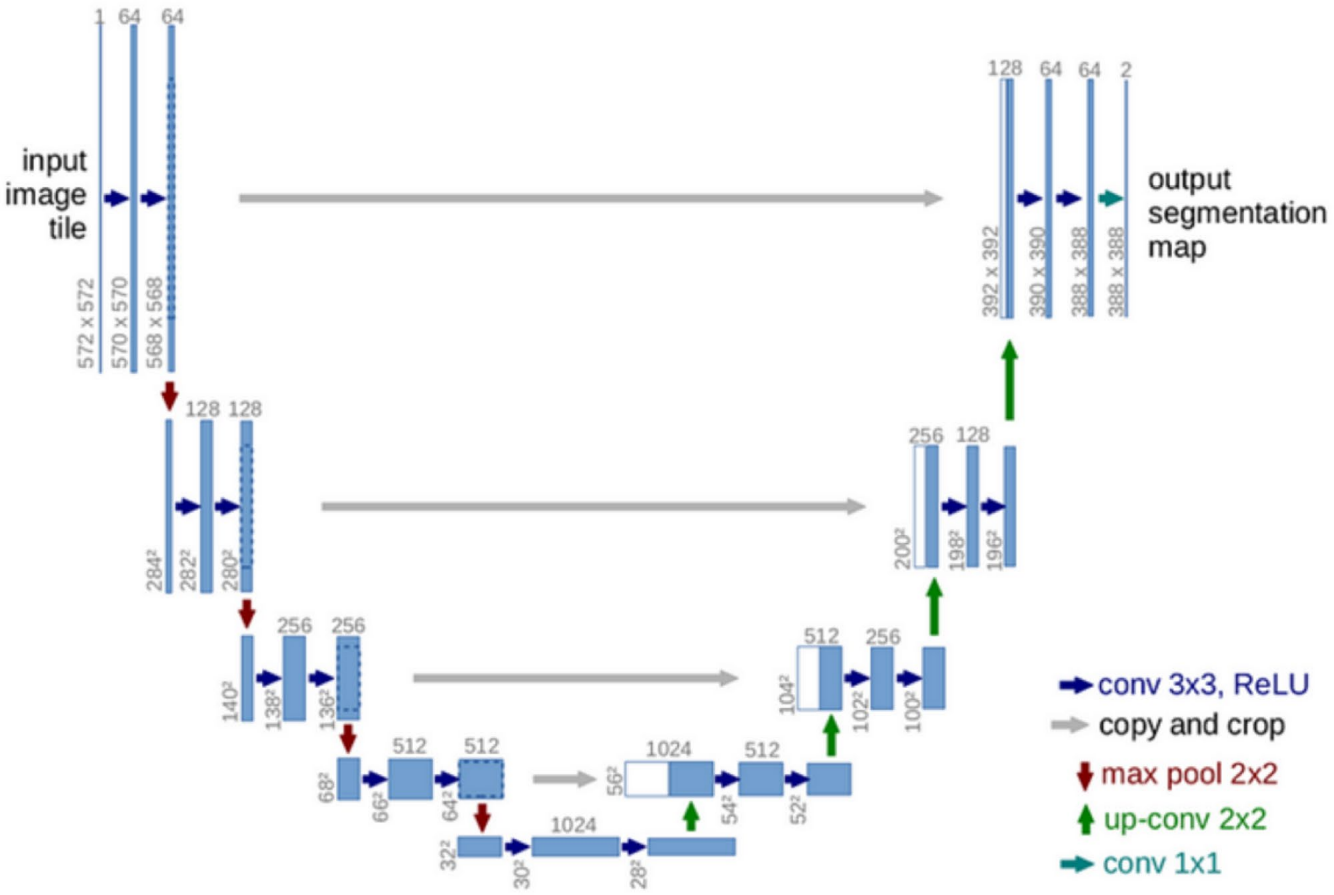
U-Net architecture.

U-Net is a fully convolutional neural network originally designed for biomedical image segmentation. It follows a symmetric encoder-decoder architecture with skip connections that efficiently combine high-level semantic features from the encoder with low-level spatial details from the decoder. The encoder path consists of multiple convolutional blocks, each containing two convolutional layers followed by batch normalization and ReLU activation. These blocks are followed by max-pooling layers that reduce spatial dimensions while increasing feature complexity. At the core of the U-Net architecture, the bottleneck layer bridges the encoder and decoder by capturing high-level semantic features with a deeper convolutional block while maintaining spatial resolution through padding. The decoder path upscales the feature maps using transposed convolutions, and each upsampling step is linked with a skip connection from the corresponding encoder layer. This strategy preserves spatial information and enables precise localization of boundaries. Finally, the output layer employs a $1\times 1$ convolution with a sigmoid activation function for binary segmentation, generating a probability map that indicates tumor regions. Figure 3 illustrates the standard U-Net architecture, highlighting the encoder, decoder, skip connections, and output layer.

###  EfficientNetB4 architecture

**Fig. 4:**
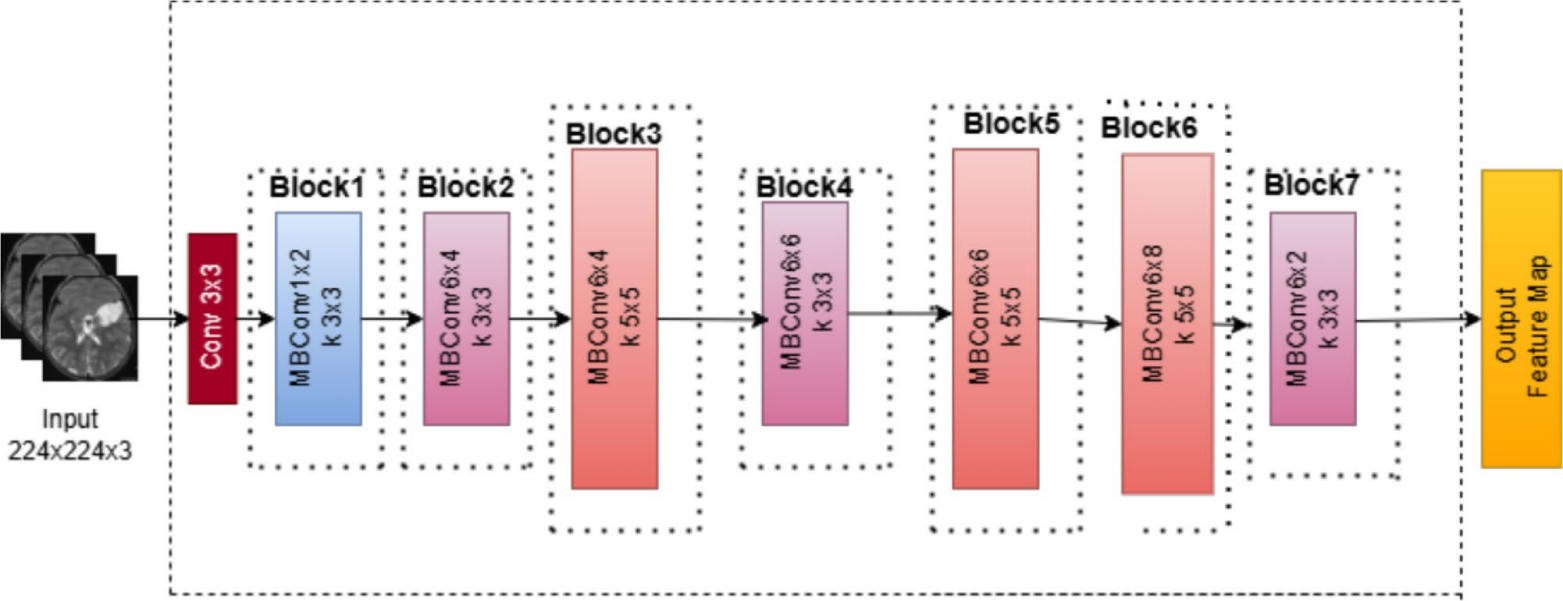
EfficientNetB4 architecture.

EfficientNetB4 is a state-of-the-art convolutional neural network designed with compound scaling that uniformly scales depth, width, and resolution, resulting in a balanced model with high accuracy and computational efficiency. It utilizes a compound scaling method that uniformly adjusts network dimensions to achieve a balance between accuracy and computational cost. This is accomplished using the Eq. (1):

1 $$\begin{aligned} d = \alpha ^\phi , \quad w = \beta ^\phi , \quad r = \gamma ^\phi \end{aligned}$$

where $d$ represents depth, $w$ is width, $r$ is resolution, and $\alpha , \beta , \gamma$ are constants determined through grid search. The architecture is built on Mobile Inverted Bottleneck Convolution (MBConv) layers, which use depthwise separable convolutions for efficient feature extraction. These blocks also incorporate Squeeze-and-Excitation (SE) modules that recalibrate channel-wise features to enhance the model’s attention to relevant features. To maintain gradient flow and prevent vanishing gradients, skip connections are integrated within the MBConv blocks. Additionally, the Swish activation function is used to improve non-linearity and overall model performance. The output layer of EfficientNetB4 generates multi-scale feature maps, which are then utilized in the U-Net decoder for precise segmentation. Fig. 4 illustrates the EfficientNetB4 architecture, detailing the flow through MBConv blocks, skip connections, and SE modules.

### Proposed multi-scale attention U-Net with EfficientNetB4 encoder

The encoder section of the proposed model leverages EfficientNetB4, a state-of-the-art convolutional neural network (CNN) architecture pre-trained on the ImageNet dataset. EfficientNetB4 was selected due to its exceptional performance and computational efficiency, which can be achieved through compound scaling technique that scales the network’s resolution, width, and depth consistently. In contrast to conventional deep learning models, this enables the model to provide high accuracy with reduced computational complexity. The EfficientNetB4 encoder is the backbone for feature extraction in the context of brain tumor segmentation. The EfficientNetB4 architecture consists of several convolutional blocks that gradually extract deeper features from the input MRI images. The input is initially processed by an initial convolution block, followed by a series of intermediate blocks that capture increasingly higher-level features required for segmentation tasks.

The encoder first passes the input image through the first convolution block, which acquires low-level image features such as edges, textures, and simple shapes to set up it for further processing. Block 2a, the second block, concentrates on moderately complex features which act as a basis for higher-level representations. Higher-level features, that give more abstract representations vital to the segmentation task, are obtained by Block3a. Block4a, a very high-level layer, further refines these features to capture more complex structures in the image. The highest-level semantic information can be found in Block6a, the deepest layer of the encoder, which focuses on the most detailed and complex representations of the input image. The decoder section of the model collects the features that were extracted from these layers and refines them using a multi-scale attention method to enhance the segmentation accuracy. For accurate brain tumor segmentation in MRI images, the model maintains high-level semantic information and fine-grained spatial details by combining EfficientNetB4’s effective feature extraction and skip connections.

The decoder section of the proposed model uses a multiscale attention mechanism to create high-resolution segmentation maps from the encoded features. By applying sequence of residual attention blocks and combining features from the EfficientNetB4 encoder with skip connections, the segmentation mask is developed. The multiscale attention mechanism makes the model ideal for segmenting complex structures like brain tumors in MRI images by improving its capacity to concentrate on relevant features at various spatial scales.

Each decoder block in the architecture functions as a residual attention block, combining multi-scale attention mechanisms with encoder skip connections to improve feature refinement. The first step involves upsampling the feature map using transposed convolutions, sometimes referred to as deconvolutions, which basically improves the feature map’s spatial resolution. In order to recover the segmented image’s finer details, this step is crucial. Convolutions with different kernel sizes (1$\times$1, 3$\times$3, 5$\times$5) are then used by the multi-scale attention mechanism to obtain contextual information at different spatial scales. This model is now able to capture features at various levels of granularity which recognizes the complex and variable nature of brain tumors. The feature maps generated by these convolutions are merged into a single feature map and passed through a 1$\times$1 convolution to match the channel dimensions. An attention mask is then produced by passing the multiscale features through another 1$\times$1 convolution and an activation layer.Through element-wise multiplication, the attention mask is applied to the feature map, allowing the model to suppress irrelevant areas and concentrate on significant areas of the image.

For medical image segmentation, feature refinement is essential, and this attention mechanism helps by highlighting the tumor areas and minimizing the background noise. After the attention operation, the resultant features are combined with the appropriate skip connections from the encoder. Fine details are preserved during the decoding process as a result to the high-resolution spatial information provided by these skip connections. A convolutional block is then used to further refine the concatenated features.

**Fig. 5:**
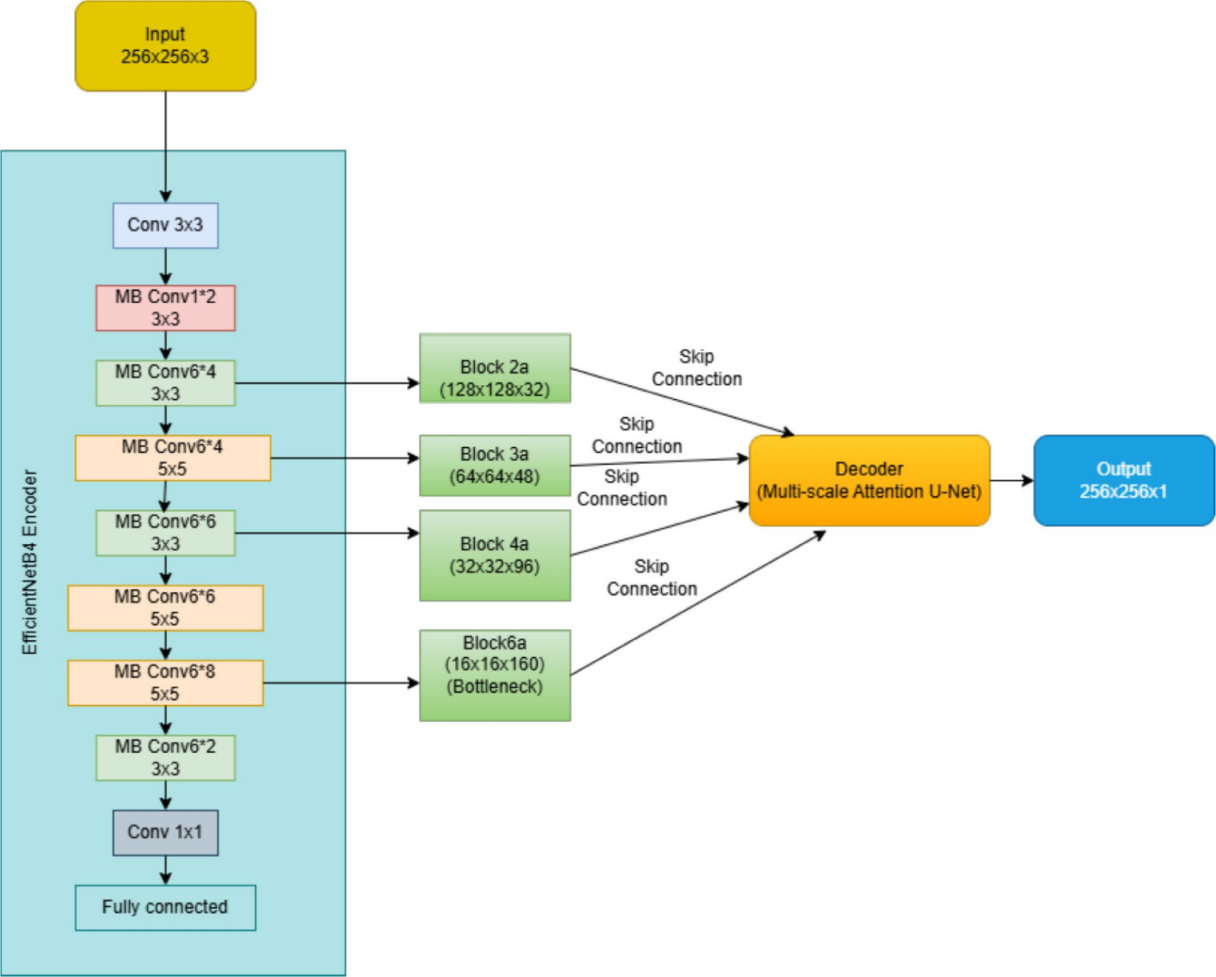
Archtecture of the proposed method.

To enhance model convergence as well as promote gradient flow during training, a residual connection has been introduced to the output of each decoder block. This residual connection helps to mitigate the vanishing gradient issue and preserves important features as the network passes through the decoder. The output of the residual connection is then enhanced with the refined features, making sure that the crucial information the network has learned is preserved. The decoder consists of a stack of these residual attention decoder blocks, where each block progressively increases the resolution of the feature map while refining the segmentation mask. The number of filters used in each block decreases as the resolution increases. The final block outputs the refined segmentation map, passing through a 1x1 convolutional layer with a sigmoid activation function to produce the final binary segmentation mask. The output of the sigmoid function represents a probability score between 0 and 1, indicating the likelihood of each pixel belonging to the tumor region, thereby achieving accurate and refined brain tumor segmentation. The proposed architecture is shown in Fig.5. This architecture combines the efficiency of EfficientNetB4 for feature extraction with the strength of the U-Net decoder and the attention mechanism to handle complex segmentation tasks, making it well-suited for precise brain tumor segmentation from MRI images.

### Mathematical formulation

The multi-scale attention mechanism is designed to capture contextual information at multiple spatial scales, enhancing the model’s ability to segment complex brain tumor structures. This is achieved by applying convolutions with varying kernel sizes, followed by an attention mask that emphasizes relevant regions.

#### Multi-scale feature extraction

Given an input feature map $X$, the multi-scale attention mechanism extracts features using different kernel sizes:

2 $$\begin{aligned} \theta _1 = W_1 * X, \quad \theta _3 = W_3 * X, \quad \theta _5 = W_5 * X \end{aligned}$$

where $W_1, W_3, W_5$ are convolution filters with kernel sizes $1\times 1$, $3\times 3$, and $5\times 5$, respectively, and $*$ denotes the convolution operation.

#### Feature concatenation

The multi-scale features are concatenated along the channel dimension:

3 $$\begin{aligned} \Theta = \text {Concat}(\theta _1, \theta _3, \theta _5) \end{aligned}$$

#### Attention calculation

To calculate the attention mask, the concatenated features are combined with a gating signal $G$ that represents the global context:

4 $$\begin{aligned} \phi _g= & W_g * G \end{aligned}$$

5 $$\begin{aligned} A= & \text {ReLU}(\Theta + \phi _g) \end{aligned}$$

6 $$\begin{aligned} \psi= & \sigma (W_\psi * A) \end{aligned}$$

where $W_g$ and $W_\psi$ are learnable weights, and $\sigma$ is the sigmoid activation function.

#### Feature refinement

The attention mask is applied to the input feature map to refine the features:

7 $$\begin{aligned} F_{out} = X \odot \psi \end{aligned}$$

where $\odot$ denotes element-wise multiplication.

## Experimental setup

This section includes the dataset details, implementation details, hyperparameter values, and performance evaluation metrics. The cornerstone of this study is the dataset selection, which includes high-quality brain MRI images for efficient training and validation. The implementation details include the software environment, libraries, and hardware specifications. Finally, a robust framework of performance evaluation metrics, including Dice Coefficient, IoU, recall, and precision, are clearly defined. Each of these components is elaborated in detail in subsequent sections to provide a clear understanding of the experimental workflow.

### Dataset

This study used 3064 T1-weighted Contrast-Enhanced Magnetic Resonance Images (CE-MRI) from 233 patients from the publicly accessible Figshare brain tumor dataset^48^. The information was gathered from China’s Nanfang Hospital, General Hospital, and Tianjin Medical University between 2005 and 2010. The dataset is organized into three categories of brain tumors: meningioma, glioma, and pituitary tumor. The original MRI images have a resolution of $512 \times 512$ pixels and are stored in MATLAB (.mat) format, which includes class labels, patient identifiers, image data, tumor boundaries, and tumor masks.

The dataset comprises a total of 3,064 brain tumor slices categorized into three classes: Glioma, Meningioma, and Pituitary Tumor. The dataset was split into 90% for training (2,758 images) and 10% for testing (306 images) to train and evaluate the proposed model. This stratified split ensures that each tumor category is proportionally represented in both the training and testing sets, facilitating robust model evaluation. The distribution of the three tumor types and data splitting ratio in the dataset is shown in Table 2. For each tumor type, images from three different planes-axial, sagittal, and coronal-are available. Sample images of each tumor type is illustrated in Fig.2

In this study, a 90:10 split was chosen for training and testing to maximize the amount of data used for model training while maintaining a reliable test set for evaluation. This approach ensures that the model learns a robust representation of tumor features from a diverse set of MRI images, leading to better generalization on unseen data. Additionally, a 10% test set provides a sufficient sample size for performance evaluation without significantly impacting the training data volume.

Cross-validation is indeed a robust method for performance evaluation, offering insights into model stability and generalization across different data subsets. However, it was not used in this study for the following reasons:

- Computational complexity: cross-validation involves training the model multiple times (e.g., 5 or 10 folds), which would substantially increase the computational cost and training time, especially given the complexity of the Multi-Scale Attention U-Net with EfficientNetB4.
- Consistency in comparison: the 90:10 split was chosen to maintain consistency with other state-of-the-art methods in brain tumor segmentation that use similar splits, ensuring a fair and direct comparison of the proposed model’s performance.
- Dataset Characteristics: The Figshare brain tumor dataset used in this study has a balanced distribution of tumor types and sufficient samples. Therefore, the 90:10 split provides reliable and statistically significant results.

To further validate the model’s robustness and generalization capability, future work could incorporate k-fold cross-validation or stratified cross-validation, particularly when extending the study to other imaging modalities or datasets with class imbalance. This would provide a more comprehensive evaluation by considering variations across different folds.

### Implementation details

TensorFlow and Keras libraries were used to achieve deep learning capabilities in the development of the proposed EfficientNetB4 with Multi-Scale Attention U-Net model in Python. The implementation was carried out on a high-performance computing environment running Windows 11 Pro and equipped with a 12th Gen Intel$\circledR$ Core$^{\textrm{TM}}$ i7-12700 processor running at 2.10 GHz, 32 GB of RAM, and an NVIDIA GeForce RTX 3070 Ti GPU. Large medical imaging datasets were trained and processed effectively due to this reliable hardware configuration. We used the Figshare repository for the dataset, which offered an extensive collection of brain MRI images along with the corresponding segmentation mask. The dataset was meticulously preprocessed to enhance segmentation performance. To reduce computational complexity and standardize input dimensions, each MRI image was resized to $256\times 256$ pixels. Images were preprocessed to the LAB color space and contrast-limited adaptive histogram equalization (CLAHE) was applied to the L-channel to enhance contrast. To ensure consistency throughout the dataset, pixel values were normalized to a range of 0 to 1 by dividing by 255 after Gaussian blur was applied to reduce noise.

The EfficientNetB4 encoder, which was chosen after an extensive analysis of all EfficientNet variants for the optimal balance between computational efficiency and feature extraction capability, is part of the model architecture. EfficientNetB4, pre-trained on the ImageNet dataset, served as the backbone for hierarchical feature extraction, capturing both low-level and high-level features essential for accurate brain tumor segmentation. The residual attention decoder blocks in the decoder section incorporate multi-scale attention mechanisms, which improve feature refinement by focusing on relevant regions at various spatial scales. This model effectively uses skip connections from the encoder to maintain spatial information and improve segmentation accuracy.

**Table 3:** Hyperparameter and values.

Hyperparameter	Value
Input image size	$256 \times 256$
Batch size	4
Number of epochs	100
Learning rate	1e−4
Optimizer	AdamW
Loss function	Dice Loss
Activation function	ReLU, Sigmoid
Early stopping patience	10
Reduce LR factor	0.1

Training was conducted over 100 epochs with a batch size of 4, utilizing the AdamW optimizer with an initial learning rate of 0.0001 and parameters $\beta _1 = 0.9$, $\beta _2 = 0.999$, and $\epsilon = 1 \times 10^{-8}$. The learning rate was dynamically adjusted using the ReduceLROnPlateau callback, reducing by a factor of 0.1 upon plateauing of the validation loss, and training was halted early with the Early Stopping callback if no improvement was observed for 10 consecutive epochs. Detailed hyperparameter settings are summarized in Table 3. The dataset was split into 90% for training and 10% for testing, ensuring a substantial portion for model evaluation. The model was compiled with a dice loss function and evaluated using metrics including accuracy, Dice Coefficient, recall, precision, Mean IoU, and IoU, providing a comprehensive assessment of segmentation performance. This meticulous implementation strategy and advanced architectural design resulted in high precision and robustness in brain tumor segmentation tasks.

### Performance evaluation metrics

Several segmentation metrics that evaluate how well predicted and ground truth segmentations match are utilized to assess the performance of the proposed model. The most commonly used evaluation metrics and their mathematical expressions are listed below.

#### Dice similarity coefficient (DSC)

A key metric to measure the overlap between the predicted segmentation (P) and the ground truth (G) is DSC. High DSC score segmentation models are utilized for accurate tumor localization, boundary detection, and treatment planning. A DSC score of 1 indicates perfect overlap. It is defined as:

8 $$\begin{aligned} \text {DSC} = \frac{2 \left| P \cap G \right| }{\left| P \right| + \left| G \right| } \end{aligned}$$

where: $\left| P \right|$: total pixels in predicted segmentation. $\left| G \right|$: total pixels in the ground truth. $\left| P \cap G \right|$: number of overlapping pixels between the predicted and ground truth regions.

#### Intersection over union (IoU)

Intersection over Union (IoU), referred to as the Jaccard Index, is a crucial metric that calculates the ratio of the intersection and union of the predicted and ground truth regions. This metric ranges from 0 to 1, where a higher value indicates better segmentation accuracy. High IoU scores support advancements in medical imaging technology by direct impact on the effectiveness of automated systems in diagnosis, treatment planning, and follow-up care.

9 $$\begin{aligned} \text {IoU} = \frac{\left| P \cap G \right| }{\left| P \right| + \left| G \right| - \left| P \cap G \right| } \end{aligned}$$

#### Mean intersection over union (mean IoU)

Mean IoU quantifies the average intersection between the predicted segmentation and the ground truth segmentation across all classes. Mathematically, it is expressed as:

10 $$\begin{aligned} \text {Mean IoU} = \frac{1}{C} \sum _{c=1}^{C} \frac{\left| P_c \cap G_c \right| }{\left| P_c \cup G_c \right| } \end{aligned}$$

where: *C* : total number of classes. $P_c$ : predicted region for class (*c*).$G_c$: ground truth region for class (*c*). $\left| P_c \cap G_c \right|$ : number of overlapping pixels between the predicted and ground truth regions for class ( *c*). $\left| P_c \cup G_c \right|$ : total pixels in the union of predicted and ground truth regions for class ( *c*).

#### Precision

Precision quantifies the ratio of accurately identified tumor pixels to the total pixels classified as tumors.

11 $$\begin{aligned} \text {Precision} = \frac{TP}{TP + FP} \end{aligned}$$

FP (False Positives) are non-tumor pixels incorrectly detected as tumors, and TP (True Positives) are correctly detected tumor pixels.

#### Recall/sensitivity

Sensitivity quantifies the ratio of precisely determined tumor pixels to the total number of actual tumor pixels. A model with a higher sensitivity is better able to detect true positive cases, which lowers the likelihood of missing real tumor instances, an essential component of timely intervention and treatment. It is calculated as:

12 $$\begin{aligned} \text {Recall/Sensitivity/TPR} = \frac{TP}{TP + FN} \end{aligned}$$

Here, TP (True Positives) are correctly detected tumor pixels, and FN (False Negatives) are tumor pixels incorrectly classified as non-tumor.

#### Specificity

Specificity assesses the capacity to identify non-tumor pixels accurately. Increased specificity reduces the likelihood of false positives, which is essential for avoiding unnecessary treatments and guaranteeing accurate diagnoses. It is determined as:

13 $$\begin{aligned} \text {Specificity/TNR} = \frac{TN}{TN + FP} \end{aligned}$$

False Positives (FP) are non-tumor pixels that are mistakenly classified as tumors, while True Negatives (TN) are non-tumor pixels that are correctly identified.

#### Accuracy

Accuracy quantifies the ratio of correctly predicted instances to the total number of instances and is calculated as:

14 $$\begin{aligned} \text {Accuracy} = \frac{TP + TN}{TP + TN + FP + FN} \end{aligned}$$

## Results and discussions

This section presents our experiment findings, focusing on the performance of the proposed EfficientNetB4 with Multiscale Attention U-Net for brain tumor segmentation. An extensive comparison is made between various EfficientNet variants, emphasizing their contributions to segmentation performance. The proposed model’s training metrics are analyzed to ensure stability and effectiveness. Finally, we compare the proposed method to cutting-edge approaches, demonstrating its superiority regarding segmentation accuracy and other key metrics.

While tumor classification is an important aspect of brain tumor analysis, our study prioritizes segmentation for several reasons. First, accurate segmentation is crucial for precise tumor localization, which directly impacts treatment planning and surgical guidance. Second, segmentation allows for better handling of tumors with irregular boundaries or overlapping intensities, which might be misclassified in direct classification approaches. Lastly, our segmentation results provide a foundation for future work in tumor classification or grading, potentially leading to more accurate and interpretable results.

### Comparison of EfficientNet variants

**Fig. 6:**
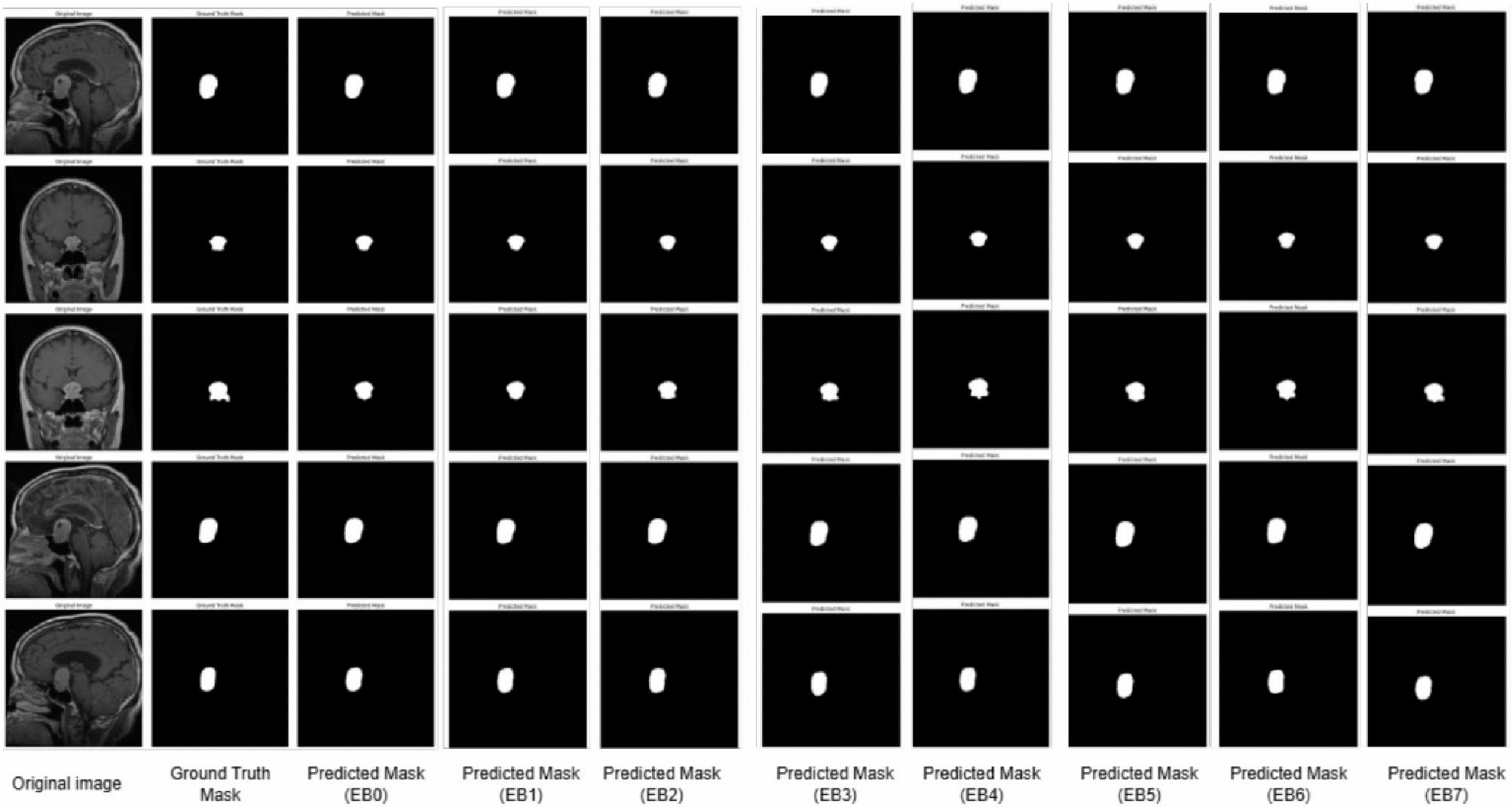
Sample images of tumor segmentation for EfficientNet variants.

Table 4 summarizes the segmentation performance of the EfficientNet variants (B0-B7) combined with the Multiscale Attention U-Net. EfficientNetB4 was the most successful backbone for our segmentation framework, achieving the highest Dice Coefficient 0.9339 and IoU 0.8795 out of all the variants. EfficientNetB4 outperformed other models in correctly recognizing tumor regions by exhibiting a balanced trade-off between feature extraction capability and parameter efficiency. These results were further supported by the segmentation outputs’ visual quality. As shown in Fig.6, the predicted masks from EfficientNetB4 closely resembled the ground truth masks, accurately capturing tumor boundaries. In contrast, other variants, such as EfficientNetB3 and EfficientNetB7, exhibited minor inconsistencies, including over-segmentation and under-segmentation, particularly in cases with complex tumor shapes. These differences highlight the importance of selecting an optimal backbone that balances model complexity and generalization ability. Interestingly, while deeper variants such as EfficientNetB6 achieved comparable performance (Dice Coefficient: 0.9327, IoU: 0.8778), they required significantly more computational resources. This makes EfficientNetB4, a more practical choice for achieving high performance with lower computational overhead.

**Table 4:** Comparison of EfficientNet variants.

Model	Dice	IoU	Precision	Recall	Specificity
EfficientNet B0	0.9127	0.8476	0.9756	0.8683	0.9998
EfficientNet B1	0.9026	0.8419	0.9738	0.8645	0.9997
EfficientNet B2	0.9289	0.8725	0.9705	0.8983	0.9997
EfficientNet B3	0.8968	0.8222	0.9864	0.8327	0.9999
EfficientNet B4	0.9339	0.8795	0.9657	0.9103	0.9996
EfficientNet B5	0.8806	0.8212	0.9647	0.8498	0.9996
EfficientNet B6	0.9327	0.8778	0.9671	0.9063	0.9997
EfficientNet B7	0.9171	0.8120	0.9817	0.8664	0.9998

### Training metrics analysis

**Fig. 7:**
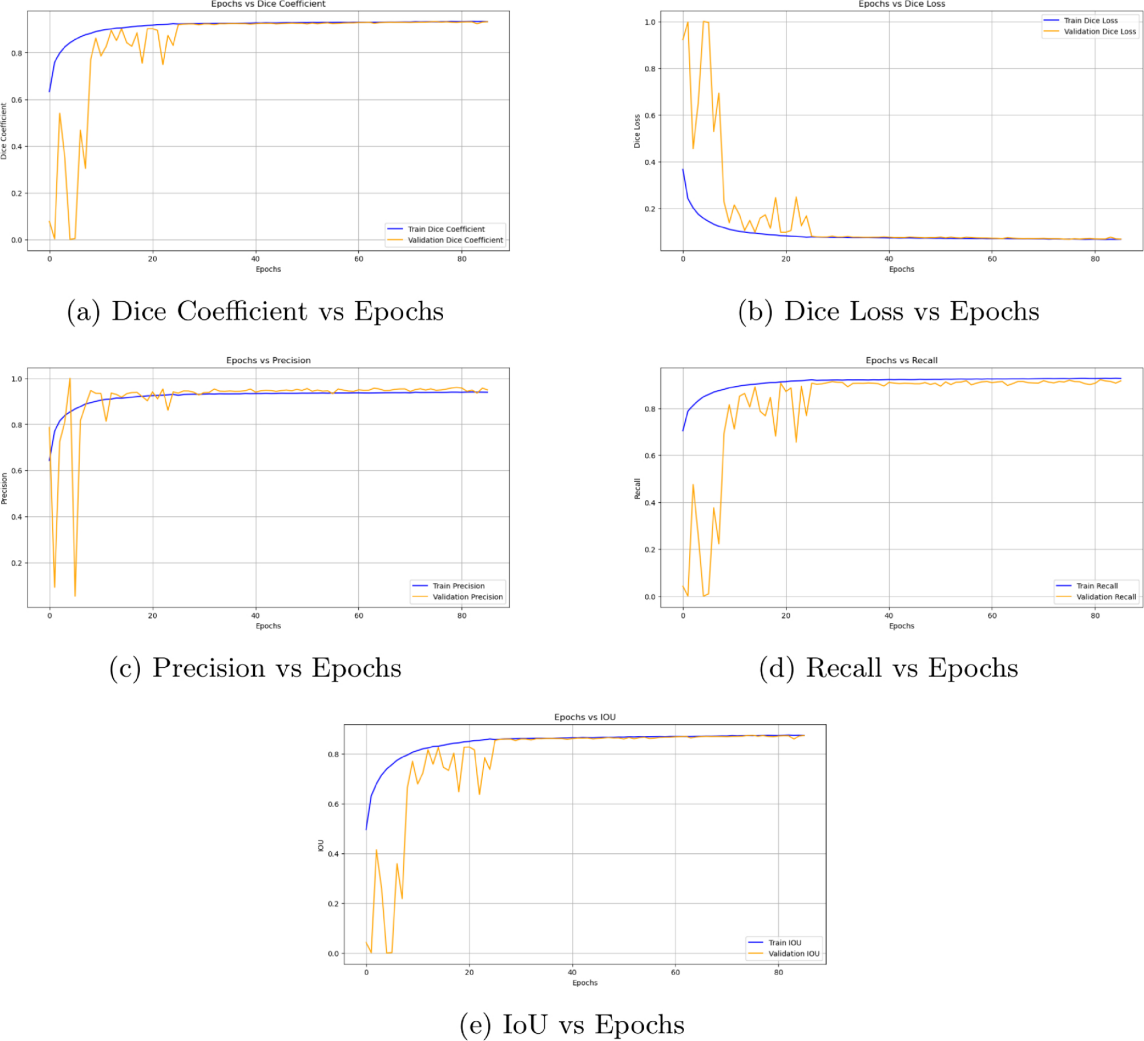
Performance metrics of EfficientNetB4 with multiscale attention U-Net monitored over epochs.

The training process of the proposed EfficientNetB4 with Multiscale Attention U-Net was monitored using key metrics, including Dice Coefficient, Dice Loss, Precision, Recall, and IoU, plotted against the number of epochs. These metrics, which are displayed in Fig. 7, offer important details regarding the stability and convergence of the model during training.

The Dice Coefficient curve increases gradually during training until it converges at 0.9339. This suggests that the model was able to continuously learn and improve its segmentation performance without overfitting. Similarly, the segmentation accuracy increased gradually, as evidenced by the Dice Loss curve’s gradual decrease. The precision and recall curves show a balanced improvement, with recall reaching 0.9103 and precision reaching 0.9657. This balance shows that the model successfully captures tumor regions without overlooking important details, while also reducing false positives. Furthermore, the model’s accuracy in localizing tumor regions is demonstrated by the IoU curve, which peaks at 0.8795. Overall, the training metrics show how robust the proposed model is, exhibiting its capacity to attain excellent performance while preserving stability and generalization.

### Visualization of misclassified images

**Fig. 8:**
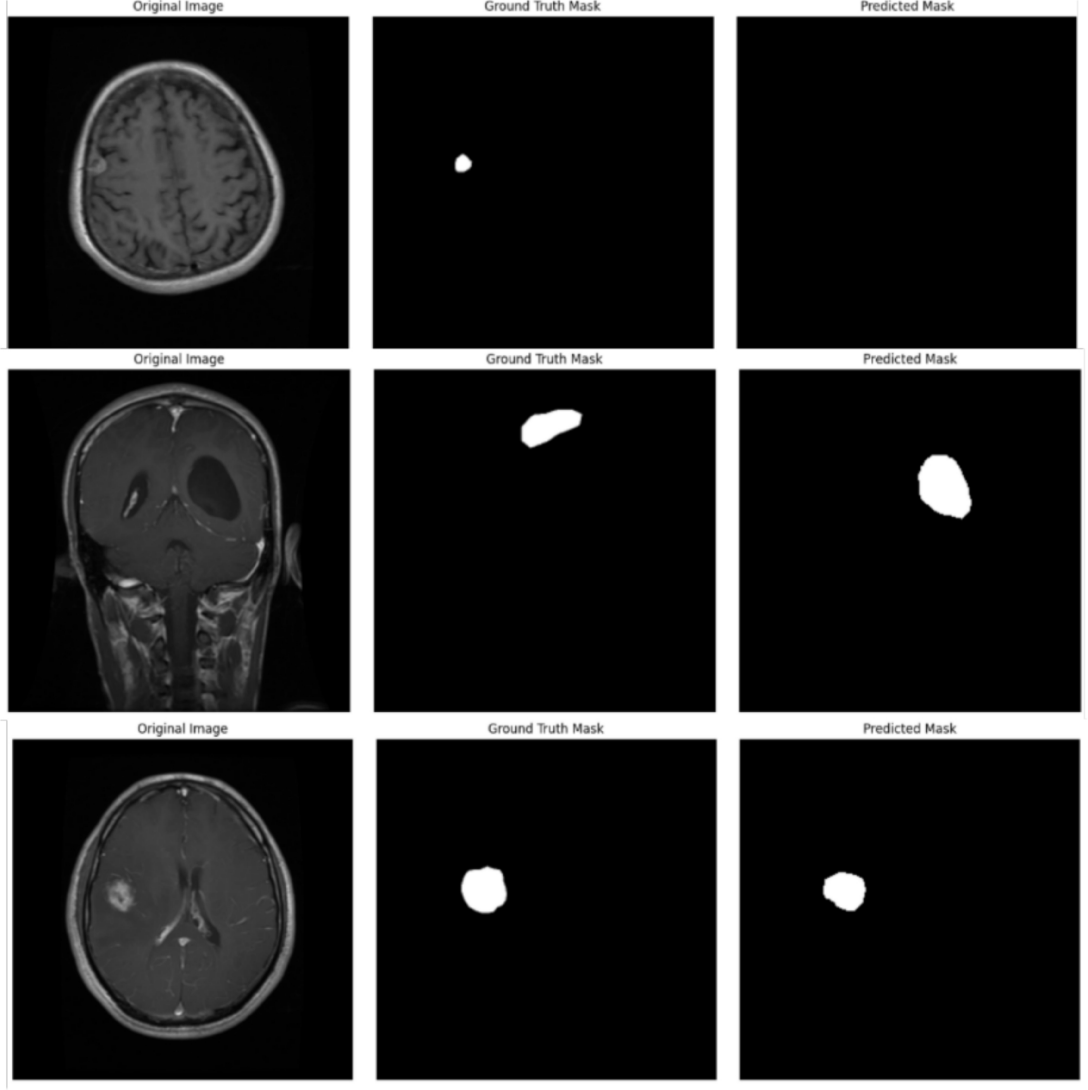
Examples of misclassified cases.

To gain insights into the limitations of the proposed EfficientNetB4 with Multiscale Attention U-Net, visualizations of misclassified cases were analyzed. Figure 8 presents examples where the model produced false positives or false negatives.

The misclassifications were primarily observed in cases with low contrast between the tumor and surrounding tissues, as well as in complex tumor shapes with irregular boundaries. These challenges resulted in false positives where non-tumor regions were segmented as tumor and false negatives where actual tumor regions were missed.

This analysis highlights the need for enhanced boundary delineation techniques and improved feature extraction methods to minimize false detections and enhance the overall segmentation performance.

### Comparison with state-of-the-art methods

The proposed EfficientNetB4 with Multiscale Attention U-Net was also compared with existing state-of-the-art methods on the publicly available Figshare brain tumor dataset. The results, shown in Table 5, reveal that our method outperformed all the compared approaches across key metrics, including accuracy, Dice Coefficient, IoU, precision, and recall. Our method achieved an accuracy of 99.79% and a Dice Coefficient of 0.9339, surpassing the best-performing state-of-the-art method by Akshya Kumar Sahoo et al.^49^. Additionally, the proposed method achieved a significantly higher IoU of 0.8795 compared to other methods such as Mayala et al. with IoU of 0.7443^50^. These improvements highlight the effectiveness of the multiscale attention mechanism in capturing tumor boundaries with greater accuracy.

EfficientNetB4’s feature extraction capabilities and the attention mechanism’s ability to concentrate on pertinent spatial features are working together to give our method its outstanding performance. The accuracy of tumor segmentation and the reduction of false detections is further highlighted by the precision value of 0.9657 and recall value of 0.9103. Furthermore, our model can generalize to a variety of tumor morphologies, as evidenced by its Mean IoU of 0.9121. These findings highlight the proposed method’s significant progress in brain tumor segmentation, establishing it as a reliable and efficient tool for clinical applications.

In contrast, previous methods showed limitations in either precision or recall, indicating challenges in maintaining consistent segmentation quality across varying tumor characteristics. For example, the methods by El-Shafai et al.^51^ exhibited lower feature extraction performance or lacked detailed Dice reporting, highlighting the advantages of the comprehensive evaluation approach of the proposed model. These findings not only showcase the proposed method’s superior performance but also emphasize its potential for clinical integration. The model’s robustness, high precision, and generalization capabilities make it a promising tool for automated brain tumor segmentation, potentially enhancing diagnostic accuracy and treatment planning. Future research could further validate these findings by testing on multi-institutional datasets and exploring its adaptability to other imaging modalities such as CT or PET scans.

In addition to accuracy and segmentation quality, inference time is a critical factor for clinical applicability. Direct comparisons of inference times with state-of-the-art models are limited in the literature. However, the proposed model achieves an average inference time of 24 ms per image, consistent with reported times for EfficientNet-based architectures used in medical image segmentation. This suggests that the model maintains a balance between high segmentation accuracy and computational efficiency, making it suitable for real-time clinical applications. Future work could involve benchmarking the model’s inference speed against contemporary architectures to provide a more comprehensive performance evaluation.

**Table 5:** Segmentation performance comparison between proposed and state-of-the-art methods on Figshare dataset.

Reference	Dataset	Accuracy(%)	Dice	IoU	Precision	Recall	Specificity	Mean IoU
^52^	Figshare	93.47	0.3518	0.2134	0.3281	0.3792	0.9619	-
^51^	Figshare	97.80	0.7600	–	0.9000	–	–	–
^53^	Figshare	–	0.8003	–	–	–	–	0.9074
^54^	Figshare	–	0.8280	–	0.9400	–	–	–
^50^	Figshare	–	0.8469	0.7443	–	0.8191	0.9985	–
^55^	Figshare	–	0.8602	–	–	–	–	–
^49^	Figshare	99.60	0.9020	–	0.9050	0.9020	0.9980	–
**Proposed method**	Figshare	**99.79**	**0.93387**	**0.8795**	**0.9657**	**0.9100**	**0.9963**	**0.91211**

Significant values are in bold.

### Statistical analysis using ANOVA test

The statistical evaluation of the proposed method was conducted using Analysis of Variance (ANOVA), focusing on the corresponding significance value. To examine the variation across multiple categories, univariate analysis of variance was employed. A One-Way ANOVA test was performed to compare the segmentation performance of the proposed method against several state-of-the-art approaches using the Figshare dataset. The results revealed a statistically significant difference among the methods (F(7, 18) = 5.0869, p = 0.0025), indicating that the proposed model outperformed the comparative approaches. However, the variance for one method could not be calculated due to the availability of a single performance metric. This limitation is acknowledged but does not affect the overall statistical significance of the findings.

The p-value was used to determine the minimum significance level for rejecting the null hypothesis. A lower p-value suggests stronger evidence supporting the alternative hypothesis, indicating higher statistical significance. For the Figshare dataset, the p-value was calculated as 0.002513, as shown in Table 6. This low p-value demonstrates the statistical significance of the proposed method’s superior performance. In ANOVA, the F-test was utilized to assess whether the variability between group means was greater than the variability within individual observations. In this context, the critical F-value (F crit) for the Figshare dataset was 2.576722, supporting the rejection of the null hypothesis. The ANOVA summary is presented in Table 6.

**Table 6:** ANOVA test on Figshare dataset.

Source of variation	SS	df	MS	F	P-value	F crit
Between groups	0.8292	7	0.1185	5.0869	0.0025	2.5767
Within groups	0.4191	18	0.0233			
Total	1.2483	25				

*SS* sum of squares, *df* degrees of freedom, *MS* mean square, *F* F-Statistic, *F crit* critical value.

### Clinical implications and deployment considerations

The high segmentation accuracy and robustness demonstrated by the proposed model suggest significant potential for clinical application in brain tumor diagnosis and treatment planning. Accurate segmentation of brain tumors is crucial for surgical navigation, radiotherapy planning, and monitoring tumor progression. However, translating this research into clinical practice requires careful consideration of regulatory and practical aspects:

1. Regulatory approvals and compliance:
  The deployment of the proposed brain tumor segmentation model as a clinical decision-support tool necessitates regulatory approval as a medical device. In the U.S., this would involve clearance or approval from the Food and Drug Administration (FDA), potentially under the Software as a Medical Device (SaMD) framework. In the European Union, compliance with the Medical Device Regulation (MDR) for CE marking is required.
  Demonstrating clinical safety, efficacy, and reliability is essential for regulatory approval. This includes validating segmentation accuracy, sensitivity, and specificity in diverse clinical scenarios.
2. Clinical validation and multi-center evaluation:
  To ensure generalizability and clinical relevance, extensive external validation using multi-center datasets with varying MRI protocols and scanner models is necessary.
  Prospective clinical studies should be conducted to assess the model’s impact on clinical decision-making, including surgical planning and treatment outcome predictions.
3. Integration with clinical workflow:
  Successful implementation depends on seamless integration with hospital systems such as Picture Archiving and Communication Systems (PACS) and Radiology Information Systems (RIS).
  The model’s interface should be intuitive, providing radiologists with easily interpretable segmentation outputs, including confidence maps and overlay visualizations on MR images.
4. Ethical, legal, and data security considerations:
  The deployment process must comply with data privacy regulations such as the Health Insurance Portability and Accountability Act (HIPAA) in the U.S. and the General Data Protection Regulation (GDPR) in Europe.
  Ethical considerations, including model transparency, accountability, and potential biases related to demographic or imaging variations, should be thoroughly evaluated.
5. User training and adoption in clinical practice:
  Radiologists and neurosurgeons require training on the model’s functionalities, interpretation of segmentation results, and understanding of its limitations.
  Continuous feedback mechanisms should be established for model updates and improvements based on real-world clinical performance.

In addition to these steps, in the future we aim to explore:

- Collaboration with regulatory bodies to streamline approval processes.
- Development of explainable AI techniques to enhance clinician trust in model outputs.
- Expansion of validation studies to include rare tumor types or atypical imaging scenarios.

By addressing these regulatory requirements and practical considerations, we aim to ensure that our proposed model is safe, effective, and ready for real-world clinical deployment.

## Conclusion

This study introduced a novel brain tumor segmentation framework that combines EfficientNetB4 with a Multiscale Attention U-Net to achieve precise and efficient segmentation on the publicly available Figshare brain tumor dataset. The proposed model achieved superior performance with an Accuracy of 99.79%, a Misclassification Rate (MCR) of 0.21%, a Dice Coefficient of 0.9339, and an IoU of 0.8795, surpassing other EfficientNet variants and state-of-the-art methods. By leveraging the multiscale attention mechanism, the model effectively captured relevant spatial features, enhancing boundary delineation and reducing false positives.

Compared to existing methods, the proposed approach demonstrated notable improvements in Dice Coefficient, accuracy, precision, recall, IoU, and mean IoU, confirming its capability to handle diverse tumor morphologies effectively. The model’s robustness was further validated through consistent convergence in key training metrics, ensuring reliable and reproducible segmentation performance. These findings establish the proposed method as a promising and efficient tool for clinical applications, particularly in brain tumor diagnosis and treatment planning. Additionally, accurate delineation of tumor regions lays a solid foundation for future research in tumor classification and grading.

## Limitations

Despite the promising results, there are several limitations to this study:

- Inference time comparison: Direct comparisons of inference times with other state-of-the-art models are limited due to the absence of standardized benchmarks. Although the proposed model’s inference time is consistent with EfficientNet-based architectures used in medical image segmentation, comprehensive evaluations against contemporary models were not conducted.
- Dataset diversity: The model’s performance was validated on the Figshare brain tumor dataset, which may not fully represent the diversity of real-world clinical scenarios. The generalization capability to other datasets or imaging modalities remains unverified.
- Multiclass segmentation: The current model is designed for binary segmentation tasks. Its performance in multiclass segmentation scenarios, such as distinguishing between different tumor types, has not been evaluated.
- Interpretability and clinical validation: While the model demonstrated high accuracy, interpretability and clinical validation were not explored in detail. This limits its immediate applicability in clinical workflows where model explainability is critical.

## Future work

To address the identified limitations and enhance the applicability of the proposed framework, future research will focus on the following aspects:

- Real-time implementation: Optimizing the model for real-time segmentation to facilitate seamless integration into clinical workflows, ensuring precise and timely tumor delineation during diagnostic procedures.
- Dataset expansion and generalization: Evaluating the model on larger and more diverse datasets, including multi-institutional data and other imaging modalities such as CT scans, to assess its robustness and generalizability across different clinical scenarios.
- Multiclass segmentation: Extending the model to handle multiclass segmentation tasks, including differentiating between gliomas, meningiomas, and pituitary tumors, to enhance its clinical utility in comprehensive brain tumor analysis.
- Domain adaptation: Implementing domain adaptation techniques to address variations in imaging protocols and scanner characteristics across different datasets, improving the model’s performance across diverse clinical settings.
- Explainable AI integration: Incorporating explainable AI techniques to provide interpretable segmentation results, enhancing clinician trust and promoting the model’s adoption in real-world medical applications.
- Clinical validation and deployment: Conducting rigorous clinical validation with expert radiologists to assess the model’s efficacy in real-world diagnostic workflows, followed by deployment trials to evaluate usability and integration challenges in clinical practice.

These enhancements aim to establish the proposed framework as a reliable, efficient, and clinically applicable tool for brain tumor segmentation, ultimately contributing to improved diagnosis, treatment planning, and patient outcomes.

## Data Availability

The dataset was gathered from the publicly available Figshare Dataset, which is available online. It is publicly accessible and unrestricted. https://doi.org/10.6084/m9.figshare.1512427 . https://figshare.com/articles/Segmenting Brain Tumors with Symmetry/1512427.
